# The dual effects of social capital on mental health: a study of structural and cognitive dimensions among the floating older adults in China

**DOI:** 10.3389/fpubh.2026.1831417

**Published:** 2026-05-08

**Authors:** Jiawen Huang, Zhuohuai Luo, Shun Yao

**Affiliations:** 1 School of Public Administration, South China University of Technology, Guangzhou, China; 2 The First Affiliated Hospital, Sun Yat-sen University, Guangzhou, China

**Keywords:** cognitive social capital, floating older adults, happiness, mental illness, neighborhood, structural social capital

## Abstract

**Introduction:**

Influenced by China's urbanization and intergenerational support culture, the population of the floating older adults in China has increased rapidly in recent years. This population faces the dual pressures of aging and mobility, resulting in a heightened risk of poor mental health. Communities are important for reconstructing social capital in resettlement areas and have rarely been considered in research on the mental health of the floating older adults. This study examined the associations of neighborhood social capital (including structural and cognitive dimensions) with the tendency toward mental illness and with happiness.

**Methods:**

The data were derived from 659 questionnaires that were completed by respondents aged 55 and above in three cities in Guangdong Province, which has relatively higher concentrations of floating older adults in China. Structural equation modeling was employed to test the relevant hypotheses in this study.

**Results:**

Although structural neighborhood social capital is positively correlated with happiness, its ability to mitigate mental illness tendency is limited. Cognitive neighborhood social capital not only significantly reduces mental illness tendency and enhances happiness but also acts as a mediating variable linking structural neighborhood social capital to mental health. Moreover, neighborhood social capital has a more significant effect on the mental health of the floating older adults with poor physical health and those who move for family care purposes.

**Discussion:**

Adopting a positive psychology perspective to define the mental health of the floating older adults, this study explores the relationship between two distinct types of neighborhood social capital and their mental health, extending the application of social capital theory to non-disaster environments. Heterogeneity analysis reveals the necessity of targeted interventions for welfare-dependent subgroups of the floating older adults.

## Introduction

The floating older adults refer to older individuals who move across districts and counties within the country for half a year or longer (1). Given the dual backdrop of increasingly severe aging and frequent population mobility, the scale of the floating older adults continues to expand in China.

According to a report on the development of the floating population in China in 2018, the number of floating older adults increased from 5.03 million in 2000 to 13.04 million in 2015. In 2020, the older adult population registered with household registration in nonnative streets reached 37.24 million (2). Due to the rapid process of urbanization and the uneven spatial allocation of public resources, the floating older adults face multiple health risks, such as the disruption of social support networks, institutional segregation, and adaptation difficulties, and have a higher incidence of mental health issues (3). Some studies have revealed that one-third of the floating older adults exhibit symptoms of anxiety or depression, with a significantly higher incidence rate than that of non-floating older adults groups (4). Focusing on the mental health research of the floating older adults not only contributes to improving the health management system for the floating population, but also holds great significance for building an older-friendly society.

In recent years, research on the mental health of the floating older adults has mushroomed. However, two limitations still need to be addressed. First, most studies define the mental health of the floating older adults from the perspective of pathology. From this perspective, mental health is regarded as a unified concept with mental illness, which constitutes a single dimension and two poles (5). Nevertheless, positive psychology opposes a focus on only the correction of negative psychological diseases and instead emphasizes the importance of cultivating positive psychological qualities (6). This perspective is rarely applied in the study of the floating older adults.

Second, although neighborhood social capital has been confirmed to be among the most critical indicators for predicting the mental health of the floating older adults, the relationship between the two is not clear (7, 8). This lack of clarity is caused mainly by the following two reasons: On the one hand, the definition of social capital in early health research was based on function rather than form (9). Many forms of neighborhood social capital, including structural social capital and cognitive social capital, have been ignored. On the other hand, everyone does not have equal access to social capital; thus, social capital does not always generate benefits (10). However, the current research does not focus on the operation of social capital, nor does it acknowledge that socioeconomic status and environmental factors play different roles in the construction of individual social capital.

To address the above gaps, this study adopts the definition of positive mental health, divides neighborhood social capital into structural and cognitive dimensions, and explores the following research questions: How do structural and cognitive neighborhood social capital influence the mental health of the floating older adults? Does cognitive social capital constitute a mediating mechanism that influences the relationship between structural social capital and mental health? Does the effect of neighborhood social capital on the mental health of the floating older adults vary by individual characteristics?

The remainder of this paper is as follows. A brief literature review is provided and research hypotheses are proposed. The data, measures, and statistical model are then described. Next, the empirical findings are reported. A discussion of the results and their implications follows. Finally, a summary of key conclusions is presented.

## Literature review and research hypotheses

### Two types of neighborhood social capital and mental health

Putnam situated social capital within the framework of communitarianism, positing that it is deeply embedded in organizational features such as trust, norms, and networks (11). As communities increasingly serve as crucial places that link social issues with individual life opportunities (9), the relationship between neighborhood social capital and mental health has garnered increasing research attention. Although there has been a lack of consensus on the concept of social capital in the field of public health research, its origins can be traced to two distinct theoretical frameworks: the network perspective and the cohesion perspective (12). The network perspective defines social capital as the collection of resources embedded within social relationships. It emphasizes the advantages that individuals can obtain through engagement in social networks or broader societal structures. The cohesion perspective regards social capital as an attribute of the group. It highlights the positive impact of the collective characteristics of social capital. On the basis of the above theoretical evolution, the classification of structural social capital and cognitive social capital has emerged. The former embodies an objective relational pattern, which is measured by individual social connections or social participation (13). The latter reflects individual deep understanding of trust, mutual support, and sharing within a special geographical context (14).

Both structural and cognitive social capital within neighborhood have been proven to be significantly correlated with mental health (15). Within a community, higher levels of cognitive social capital are associated with improved mental health among older adults. Reciprocity among neighbors is negatively correlated with depressive tendencies of older adults (16). Familiarity with community members, trust in them, and a general sense of community safety promote their mental health, such as happiness and cognitive function (17). This effect is particularly pronounced in rural areas (18). In comparison, the relationship between structural social capital and mental health is more complex (19). Social connections with community residents alleviate the impact of adverse events later in life and reduce loneliness (20). Residing in a community characterized by active social interaction prevents social isolation and a lack of social support (21). However, some studies also propose that the relationship between structural social capital and the mental health of older adults may become irrelevant (22) or even have negative effects (23). Because obligations embedded in social networks within communities may become a source of personal pressure.

Shaped by their early-life experiences and the influence of their hometowns, the mindset and behavioral patterns of the floating older adults tend to be deeply rooted (24). Changes such as relocation and aging in later life easily lead to adaptation and psychological health issues for them (25). Combine previous theoretical viewpoints, this study points out that both structural and cognitive neighborhood social capital play important roles in enhancing the mental health of the floating older adults, but their impact pathways are different.

The role of structural neighborhood social capital lies in the establishment of social relationships and the acquisition of resources. On the one hand, the floating older adults swiftly reconstruct social support networks to access a broader range of material, emotional, and informational resources by neighborhood interaction in the relocation destination (25), thus reducing negative emotions stemming from stress. Active participation in community activities is also important for promoting communication between the floating older adults and local residents (26), which facilitates acceptance of local cultural customs and new lifestyles (27), enabling them to experience positive emotions. Therefore, structural neighborhood social capital primarily promotes the mental health through resource compensation and behavioral integration.

The impact of cognitive neighborhood social capital is primarily manifested in the reconstruction of identity and subjective awareness. Mutual assistance and trust among neighbors enable the floating older adults to associate the positive attributes of those around them with perceptions of the new community, thus fostering a sense of belonging (28). A community atmosphere marked by harmony and tolerance also helps them integrate into urban life with a fair and respectful attitude, and strengthens the sense of self-identity (27). These positive functions generated by cognitive social capital alleviate their negative emotions and nurture a optimistic outlook on life. Based on the above point of view, this study puts forward the following hypotheses:

Hypothesis 1: Structural neighborhood social capital is significantly positively related to mental health.

Hypothesis 1.1: Structural neighborhood social capital is significantly negatively related to mental illness tendency.

Hypothesis 1.2: Structural neighborhood social capital is significantly positively related to happiness.

Hypothesis 2: Cognitive neighborhood social capital is significantly positively related to mental health.

Hypothesis 2.1: Cognitive neighborhood social capital is significantly negatively related to mental illness tendency.

Hypothesis 2.2: Cognitive neighborhood social capital is significantly positively related to happiness.

During the analysis of the two forms of social capital, cognitive social capital has been verified to be the mediating factor in the relationship between structural social capital and mental health, with this particular conclusion centered mainly on disaster scenarios. The cognitive facet of social capital, emerging as an outcome of its structural counterpart, affects the depressive inclinations and life satisfaction of those affected by disasters (19). This causal sequence, on the one hand, elucidates the vague and fragile link between structural social capital and mental health (28). On the other hand, it emphasizes the different roles of these two forms of social capital. Structural social capital provides essential resources, while cognitive social capital plays an important role in fostering a favorable environment (29). Indeed, it is precisely that structural social capital serves as a prerequisite for the formation and activation of cognitive social capital that community members bolster their sense of control over their lives and the surrounding environment through concerted collective efforts, consequently mitigating mental health challenges (30). This study posits that such a mediating effect may similarly extend to the floating older adults. The floating older adults acquire resources and opportunities via neighborhood interactions and community engagement (11). These forms of structural social capital are further transformed into favorable assessments of the community, including feelings of trust and mutual support, thus fostering a more optimistic mental health outlook. Therefore, this study puts forward the following hypotheses:

Hypothesis 3: The positive effect of structural neighborhood social capital on mental health is mediated by cognitive neighborhood social capital.

Hypothesis 3.1: The negative effect of structural neighborhood social capital on the tendency of mental illness is mediated by cognitive neighborhood social capital.

Hypothesis 3.2: The positive effect of structural neighborhood social capital on happiness is mediated by cognitive neighborhood social capital.

### Heterogeneity of neighborhood social capital and mental health

The impact of neighborhood social capital on mental health of older adults varies according to different social scenarios, which include both the characteristics of communities and individuals (31). Among these factors, age, income, and education stand out as the most frequently discussed dimensions in heterogeneity analysis (32, 33). Compared with other older adults, the floating older adults have two characteristics, namely, aging and mobility, which serve as the basis for this study to discuss heterogeneous effects.

Physical health, as one of the indicators for heterogeneous analysis, reflects the aging attribute of the floating older adults. Physical health decline is not only a natural process of aging but also profoundly reshapes individuals' dependence patterns on social resources. As they age, older adults's range of activities gradually becomes limited, and their scope of daily life is often within their residential community (34). This spatial constraint substantially heightens their reliance on community resources (35), resulting in a greater impact of neighborhood social capital on their life satisfaction (29).

For the floating older adults, the aforementioned impacts are particularly prominent. neighborhood environment is an important place to reestablish order in their life and experience social integration. However, poor health weakens their ability to actively communicate and establish relationships in their new community, thereby limiting the space of their social network. In this context, the resource substitution theory points out that the resources obtained by embedding in a single network increase personal dependence on such specific resources (36). Without alternative choices, the floating older adults rely more heavily on the effectiveness of neighborhood social capital for their subjective welfare and mental health (37).

Moreover, the positive effect of social capital on health is more prominent when individuals are facing high pressure (38). For floating older adults with poor physical health, discomforts such as physical pain and functional decline exacerbate their stress burden, highlighting the positive effect of neighborhood social capital on their mental health. Based on the above discussion, this study puts forward the following hypotheses:

Hypothesis 4: Neighborhood social capital has a stronger effect on the mental health of the floating older adults with poorer physical health.

Hypothesis 4.1: Structural neighborhood social capital has a stronger negative effect on the mental illness tendency of the floating older adults with poorer physical health.

Hypothesis 4.2: Structural neighborhood social capital has a stronger positive effect on the happiness of the floating older adults with poorer physical health.

Hypothesis 4.3: Cognitive neighborhood social capital has a stronger negative effect on the mental illness tendency of the floating older adults with poorer physical health.

Hypothesis 4.4: Cognitive neighborhood social capital has a stronger positive effect on the happiness of the floating older adults with poorer physical health.

Mobility, as a crucial attribute of the floating older adults, serves as another variable for heterogeneity analysis in this study. It refers to the relocation of individuals across regions between countries or in different parts of the country (39). Affected by many factors, such as the place of origin, personal and family characteristics, mobility can be categorized into various types, with the purpose of mobility serving as one of the most important criteria for classification (40). In developed countries, the floating older adults usually move to other countries in search of political asylum, because of a technical assignment or to improve their quality of life (41). Compared with these kinds of international mobility, the floating older adults in China are defined as older adults population who have moved across districts and counties within China for at least 6 months. They can be divided into two main types, family care workers and migrant workers (1), which exhibit distinct patterns of social interaction, thereby potentially shaping their engagement with neighborhood social capital.

The main reason for mobility among the family-care-oriented floating older adults is to provide family support for their children, including undertaking housework and taking care of their children and grandchildren's daily lives. Since they devote most of their time and energy to their families, they are more likely to be embedded in the community environment where the family is located (42). They are also active users of community public spaces (8), are willing to exchange childcare experiences with community-dwelling older adults and even participate in various sports activities and interest organizations. Such engagement embeds the family-care-oriented floating older adults in local social networks, making the community a key determinant of their quality of life, including mental health.

In contrast, the purpose of older migrant workers is to maintain the livelihood of their families. They generally experience mobility at a young age. As they grow older, they continue to live and work locally or move to other places (43). Rich mobility experience makes them more adaptable and able to make new friends in unfamiliar environments. Moreover, because they are still working, the two living spaces including the workplace and the community, provide them with opportunities to build diversified social networks and reduce their dependence on community relationships. Some studies have shown that the number of local friends made by older migrant workers at the place of relocation is higher than that of the family-care-oriented floating older adults (44). These differences suggest that neighborhood social capital may play distinct roles in shaping the mental health of the two groups. This study therefore proposes the following:

Hypothesis 5: Neighborhood social capital has a stronger effect on the mental health of family-care-oriented floating older adults.

Hypothesis 5.1: Structural neighborhood social capital has a stronger negative effect on the mental illness tendency of family-care-oriented floating older adults.

Hypothesis 5.2: Structural neighborhood social capital has a stronger positive effect on the happiness of family-care-oriented floating older adults.

Hypothesis 5.3: Cognitive neighborhood social capital has a stronger negative effect on the mental illness tendency of family-care-oriented floating older adults.

Hypothesis 5.4: Cognitive neighborhood social capital has a stronger positive effect on the happiness of family-care-oriented floating older adults.

**Figure 1:**
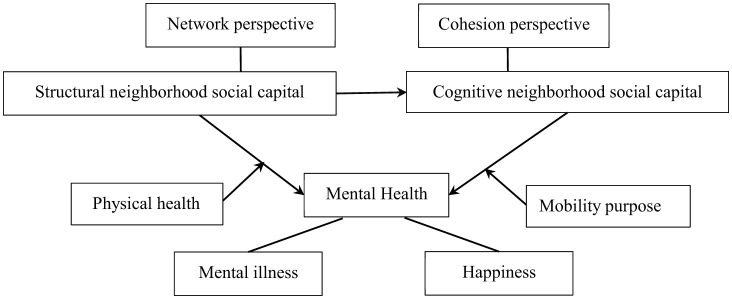
Analytical framework of this study.

The analytical framework of this study is shown in Figure 1.

## Materials and methods

### Sampling

The data in this study were sourced from a questionnaire survey conducted from May to August 2022 in three cities in Guangdong Province, which has relatively higher concentrations of floating older adults in China. The survey adopted quota sampling. The specific steps were as follows: First, three cities, including Guangzhou, Shenzhen, and Foshan, were selected as the survey cities based on the proportions of older adult population and floating population in the total municipal population according to the Seventh Population Census. Second, within each city, the survey randomly selected two streets. From each of these streets, two communities were chosen. The Human Resources and Social Security Department of Guangdong Province contacted the community head of the selected community to facilitate the access for the survey. Third, community staff recruited eligible floating older adults via WeChat groups and phone calls. To meet the sample size requirements of the statistical model and ensure statistical power, this study followed the recommendations of Bentler and Chou (45) and aimed to recruit 55 respondents per community. With the informed consent of the interviewees, trained interviewers conducted face-to-face structured interviews with them at their homes or in public community spaces.

Eligible participants met the following criteria: (1) aged 55 or above. The age of 55 was set as the threshold based on two considerations: for women, it is the statutory retirement age in China; for men, reaching 55 marks entry into the “quasi-older” phase, with physical, mental, and behavioral traits resembling those of older adults, thereby prompting contemplation of later-life issues (46). (2) nonlocal residents who have resided in the survey city for more than 6 months.(3) have normal cognitive function and hearing ability. The size of the final sample was 659, with complete information on important demographic variables.

### Measurement

#### Dependent variable

This study adopted the positive psychology perspective to define mental health, which referred to a state of flourishing without mental illness (6). In accordance with the criteria of the fifth edition of the Diagnostic and Statistical Manual of Mental Disorders (DSM-5), mental illness was assessed based on whether the following four symptoms had been present during the past 2 weeks: “I feel depressed or helpless,” “I am not interested in anything,” “I feel nervous, anxious or irritable,” “I feel scared or frightened”. The answers included: 1 = not at all; 2 = not more than 2 days; 3 = lasting for 3–4 days; 4 = lasting for 5–9 days; and 5 = lasting for 10–14 days. Each item was coded as 1 to 5, representing the severity of mental illness tendency. The results of Cronbach's alpha (0.763) and KMO (0.761) revealed that the four items had internal consistency. In statistical analysis, these items were named mi1~mi4.

Drawing on the approach of Keyes and Simoes (6), this study utilized Keyes's scales for emotional and social wellbeing, along with Ryff's scales for psychological wellbeing, to assess happiness. Emotional wellbeing included the following five items: “being able to maintain a good mental state,” “feeling happy,” “feeling peaceful/calm,” “feeling energetic” and “being satisfied with life”. Psychological wellbeing included the following seven items: “I like my personality,” “I can easily build trust with others,” “life is a process of continuous learning and change,” “life enrichment,” “my goal is clear,” “I am good at managing daily life” and “I am not easily influenced by others”. Social wellbeing included the following five items: “people care about the difficulties encountered by others,” “society is constantly improving my situation,” “I have made contributions to the community,” “I understand what is happening in the world” and “I have a close relationship with others in the community”. The answer for each item ranged from 1 to 5. The higher the score is, the higher the level of happiness. The KMO values of the three scales were 0.837, 0.793 and 0.703, respectively, and the Cronbach's alpha values were 0.836, 0.714 and 0.701, respectively. The above results indicated that the measurement of happiness was applicable in this study.

#### Independent variables

Currently, there is a lack of unified standards for measuring structural and cognitive neighborhood social capital. Drawing on existing literature, structural neighborhood social capital refers to the observable social connections between individuals and community members, and is generally divided into two aspects: social networks with neighbors and community participation (47). In this study, the former was measured by four items: the number of residents with whom the respondent meets and greets; the number of residents whom the respondent can visit at home; the number of close friends; and the number of ordinary friends within the community. The latter was measured by the frequency of participation in various activities organized by the community. The answer of each item was assigned a score ranging from 1 to 5. In the statistical analysis, these items were named ssc1~ssc5.

Cognitive neighborhood social capital refers to personal subjective evaluation of the trust, shared norms and values generated by the network within the neighborhood, which is measured by trust, mutual assistance and a sense of belonging (47). The following five items were used for measurement: “the residents within the community are trustworthy,” “the people within the community are willing to help each other,” “I can borrow what I need from the neighbors,” “I feel at home in the community,” and “the people in the community can respect each other”. The answer of each item was assigned a score ranging from 1 to 5. In the statistical analysis, these items were named csc1~csc5. The KMO values of the two types of neighborhood social capital were 0.806 and 0.743, respectively, and the Cronbach's alpha values were 0.841 and 0.701, respectively, indicating that the scale has internal consistency.

#### Other variables

Gender, age, marital status, education, household registration, physical health status and income were included in the model analysis. The selection of control variables was based on these considerations. Age increases the risk of adaptation difficulties and social isolation for the floating older adults (48). Having a spouse can buffer loneliness and cultural pressure; and its alleviating effect on loneliness in older men is stronger than that in older women (49). The floating older adults with higher income, physical health, and education are more likely to maintain their mental health by rebuilding social networks (50). Registered residence impacts the mental health by shaping their adaptability to urban living and the distribution of public resources (51). Gender, marital status and household registration were categorical variables with the following codes: 0 = male, 1 = female; 0 = single, 1 = nonsingle; 0 = agricultural registered permanent residence, 1 = nonagricultural registered permanent residence. Education (1 = primary school and below, 2 = junior school, 3 = senior high school, 4 = university or above) and physical health status (1 = unhealthy, 2 = generally healthy, 3 = healthy) were recorded as ordered variables. Age and income were numeric variables.

### Data analysis

Data analysis was conducted using Stata 16.0. This study initially employed structural equation modeling (SEM) to examine the direct impact of structural neighborhood social capital and cognitive neighborhood social capital on mental health. In the definition of positive mental health, mental health can be divided into mental illness and happiness, which constitute distinct continua rather than opposite ends of a single spectrum (6). In this study, the methods of Keyes and his collaborators (6) were used to determine the correlation between mental illness and happiness through SEM. After the main control variables were incorporated, the variable information matrix was used to calculate the standard error. SEM was considered to have an acceptable fit based on the following criteria (52): X2/df <3, RMSEA <0.08, CFI > 0.90, TLI>0.90, and SRMR <0.08.

Second, the role of cognitive neighborhood social capital as a mediating variable of the relationship between structural neighborhood social capital and mental health was investigated. To address the limitations of the BK test and fulfill the normal distribution requirement of the Sobel test, the BK modified method and the Monte Carlo method were adopted to reexamine the mediating effect.

Finally, this study used multigroup structural equation modeling to test the heterogeneous effects of neighborhood social capital on mental health based on different levels of physical health and the purpose of mobility. The analysis included four sequential steps (53): (1) Pass the measurement invariance test, which means achieving at least the second level of invariance (metric invariance). (2) A baseline model with no equality constraints on parameters across subgroups was specified. (3) The chi-square of each nested model, which is a constrained key path, is compared with the baseline unconstrained model. A significant difference in the chi-square test compared with the baseline model indicated that the corresponding parameters differed between each subgroup. (4) According to step 3, the final model retains only the equality constraints, which do not cause a significant change in the chi-square value compared with the baseline model.

## Results

The measurement model of mental health shows that X2/df = 2.507, RMSEA = 0.049, CFI = 0.988, TLI = 0.977, SRMR = 0.027. The measurement model of neighborhood social capital displays that X2/df = 2.239, RMSEA = 0.044, CFI = 0.983, TLI = 0.973, SRMR = 0.040. The indices indicate a high degree of fit between the data and the measurement models. The standardized estimates of the factor loadings of mental illness and happiness ranged from 0.558 to 0.816 and 0.535 to 0.892, respectively. The factor loadings ranged from 0.608 to 0.796 for structural neighborhood social capital and from 0.511 to 0.568 for cognitive neighborhood social capital.

### The direct effects of structural and cognitive social capital on mental health

**Figure 2:**
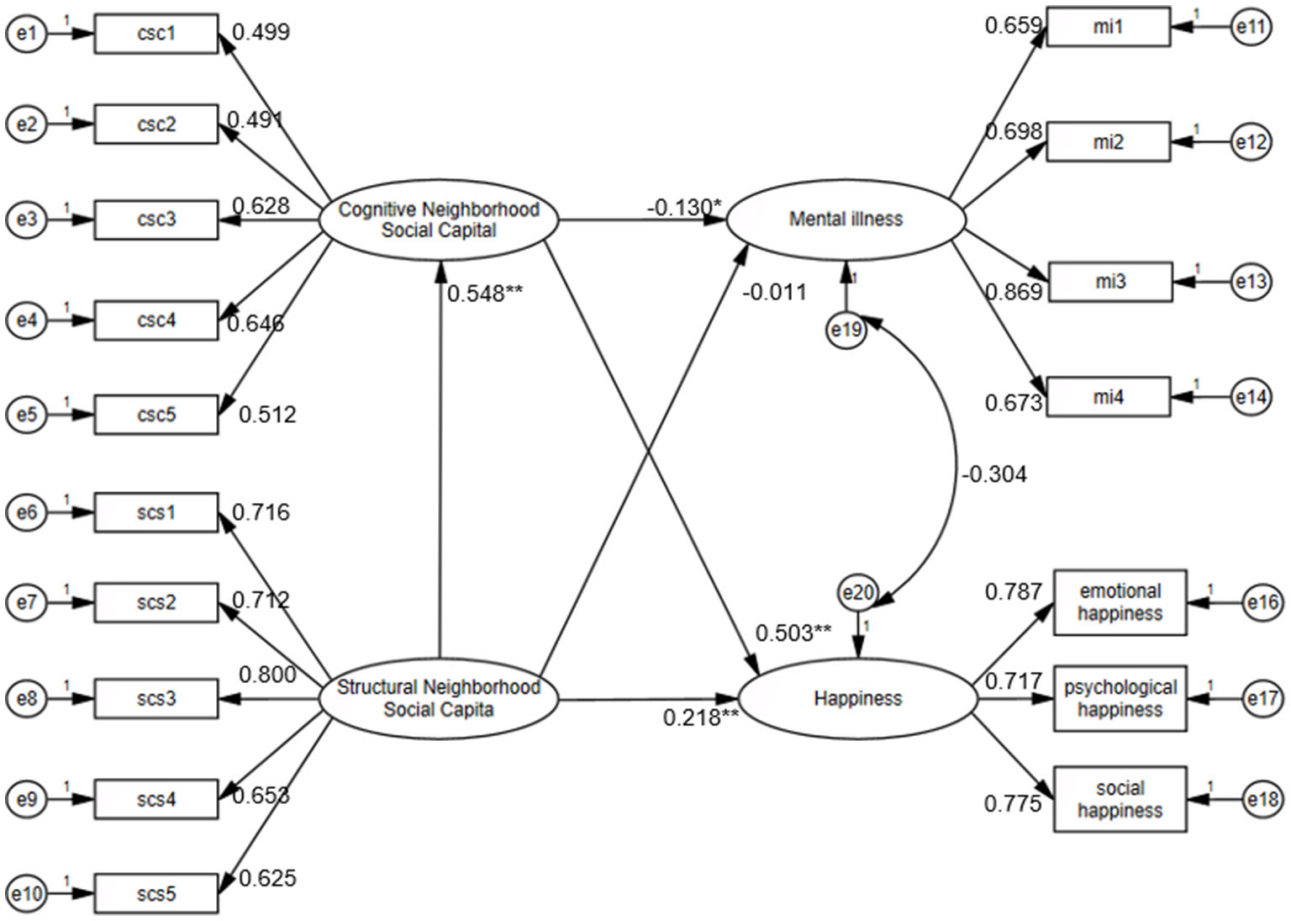
The relationship between neighborhood social capital and mental health. Note. Standardized coefficients are reported. *, ** indicate significance at the 5% and 0.1% levels (two-tailed), respectively. Gender, age, marital status, education, household registration, physical health status and income were incorporated into the statistical analysis as control variables.

In this study, SEM was used to examine the direct effects of two types of neighborhood social capital on the mental health of the floating older adults. Some main demographic and social characteristics were included in the models. The results are shown in Figure 2. The model fit indices met the standard: X2/df = 2.177, RMSEA = 0.043, CFI = 0.938, TLI = 0.921, and SRMR = 0.046.

Structural social capital had a significant positive effect on happiness (beta = 0.218, *P* < 0.001), but its effect on mental illness was not significant. The results indicated that higher structural social capital effectively enhanced the happiness of the floating older adults but did not alleviate their mental illness. As a result, hypothesis 1.2 was supported by the data, whereas hypothesis 1.1 did not match the results. While structural neighborhood social capital provides low-cost emotional benefits that boost happiness among the floating older adults, superficial interactions like greetings and chats cannot ease their psychological isolation and sense of meaninglessness caused by feeling like outsiders in the host community due to cultural differences.

Cognitive social capital was significantly positively associated with happiness (beta = 0.503, *P* = <0.001) and significantly negatively associated with mental illness tendency (beta = −0.130, *P* < 0.05). These findings indicated that higher levels of cognitive social capital were associated with both higher levels of happiness and lower levels of psychological distress among the floating older adults. The results above fully support hypotheses 2.1 and 2.2. The reciprocal support, interpersonal trust, and sense of community belonging arising from newly established social relationships in the relocation destination serve to reshape the subjective identity and community perception of the floating older adults, thus promoting their mental health.

### The cognitive mediation of structural social capital and mental health

Figure 2 also shows that structural neighborhood social capital had a significant positive effect on cognitive neighborhood social capital (beta = 0.548, *P* < 0.001). Based on the results indicating that cognitive social capital had a significant impact on both dimensions of mental health, this study finds that structural social capital reduces the tendency of floating older adults toward mental illness and enhances their sense of happiness by promoting cognitive social capital.

To further examine the mediating role of cognitive social capital, this study used the BK-modified method and the Monte Carlo method to conduct additional tests on the mediating effects. As presented in Table 1, the Sobel test statistics of cognitive social capital were−2.031 (*P* < 0.05) and 7.661 (*P* < 0.001), respectively. The Monte Carlo statistics of cognitive social capital were−2.015 (*P* < 0.05) and 7.640 (*P* < 0.001), respectively. The path coefficients for a and b were statistically significant, but the direct effect of structural social capital on mental illness was not significant, whereas its direct effect on happiness remained significant (c′-path). These findings suggest that cognitive social capital plays a complete mediating role between structural social capital and mental illness and a partial mediating role between structural social capital and happiness. In conclusion, the SEM, the BK-modified method and the Monte Carlo method jointly proved hypothesis 3.1 and hypothesis 3.2. Structural neighborhood social capital is a fundamental prerequisite for shaping mental health. It acts as a key catalyst for transformation by fostering the redefinition of roles and a sense of identity and belonging within the community, ultimately generating dual positive impacts on mental health.

**Table 1:** Results of the mediation effect test.

Independent variable	Structural neighborhood social capital	
Mediating variable	Cognitive neighborhood social capital	
Dependent variable	Mental illness	Happiness
a	0.548^***^	0.548^***^
b	−0.130^*^	0.503^***^
c'	−0.011	0.218^*^
c	−0.049	0.481^***^
m	−0.071^*^	0.276^***^
Sobel	−2.031^*^	7.661^***^
Monte carlo	−2.015^*^	7.640^***^
RIT	(0.071/0.083) = 0.862	(0.276/0.493) = 0.559
RID	(0.071/0.011) = 6.261	(0.276/0.218) = 1.267

a denotes the path coefficient between the independent variable and the mediating variable.

b denotes the path coefficient between the mediating variable and the dependent variable.

c denotes the path coefficient between the independent variable and the dependent variable (without the mediating variable included).

c′ denotes the path coefficient between the independent variable and the dependent variable after the mediating variable is included.

Sobel is a statistic used to test whether the mediation effect (a × b path coefficients) is significantly different from zero. Monte Carlo refers to the statistical value obtained by the Sobel calculation method after several simulations. RIT represents the proportion of the mediating effect in the total effect. RID represents the ratio of the mediating effect to the direct effect.

^*^, ^**^, and ^***^ indicate significance at the 5%, 1%, and 0.1% levels, respectively.

### Heterogeneous effects of neighborhood social capital on mental health

Based on the two attributes of aging and mobility of the floating older adults, this study selected physical health and mobility purpose as variables for heterogeneous analysis. After following the four steps of multigroup structural equation modeling, the results were as follows. On the basis of the invariance criteria, the floating older adults were divided into two groups, namely “low physical health” and “high physical health,” and then incorporated into structural equation modeling. The results presented in Table 2 show that after imposing equal constraints on mental illness and happiness, the change in the chi-square coefficient was only significant for the path from structural neighborhood social capital to happiness.

**Table 2:** Fit index of models of structural neighborhood social capital with equality constraints of the main pathway coefficients based on the level of physical health.

Paths	*x* ^2^	df	ΔX^2^ from the base model	Δx^2^, *P* =	RMSEA	CFI	SRMR
1.Unconstrained base model	431.760	236	–	–	0.051	0.933	0.055
*Constrained paths*							
2. S_CSC → Mental illness	431.930	237	0.17	0.680	0.051	0.933	0.055
3. S_CSC → Happiness	438.850	239	7.09	0.008^*^	0.052	0.931	0.057

Standardized coefficients are reported.

^*^ indicates significance at the 1% level (two-tailed).

Gender, age, marital status, education, household registration and income were incorporated into the statistical analysis as control variables.

Table 3 indicates that on the basis that the fitting index meets the standard, there were significant differences in the effect of structural neighborhood social capital on happiness among the different levels of physical health. The positive effect of structural neighborhood social capital on the happiness of floating older adults with low levels of physical health (beta=0.606, *P* < 0.001) was higher than on those with high levels of physical health (beta=0.485, *P* < 0.001). Hypothesis 4.2 was thus proven, whereas hypothesis 4.1 was not.

**Table 3:** Results of structural equation modeling of structural neighborhood social capital on different levels of physical health.

Model	Variable relationship	Levels of physical health	
		(Standardized coefficient)	
		Low level of physical health	High level of physical health
Structural model	Structural neighborhood social capital → Mental illness	−0.051	−0.108
	Structural neighborhood social capital → Happiness	0.606^*^	0.485^*^

Standardized coefficients are reported.

^*^indicates significance at the 0.1% level (two-tailed).

Gender, age, marital status, education, household registration and income were incorporated into the statistical analysis as control variables.

As shown in Table 4, after imposing equal constraints on mental illness and happiness, the change in the chi-square coefficient was significant for the path from cognitive neighborhood social capital to mental illness tendency but not significant for the path to happiness. Table 5 shows the alleviating effect of cognitive neighborhood social capital on mental illness tendency existed only among floating older adults with low levels of physical health (beta = −0.191, *P* < 0.01). There was no significant difference in the positive effect of cognitive neighborhood social capital on happiness between the two levels of physical health. Hypothesis 4.3 was thus supported by the results, whereas hypothesis 4.4 was not. Declining physical health imposes multiple physiological and disease management pressures on the floating older adults while simultaneously restricting their daily activity radius, thereby rendering the community their primary social space. As such, structural social capital boosts their happiness through immediate emotional interactions. Meanwhile, mental illness tendencies, rooted in deep alienation, depend more on cognitive social capital to be relieved by reshaping intrinsic meaning perception.

**Table 4:** Fit index of models of cognitive neighborhood social capital with equality constraints of the main pathway coefficients based on the level of physical health.

Paths	*x* ^2^	df	Δx^2^ from the base model	Δx^2^, *P* =	RMSEA	CFI	SRMR
1. Unconstrained base model	367.220	244	–	–	0.040	0.939	0.051
*Constrained paths*							
2. R_CSC → Mental illness	373.160	245	5.94	0.015^*^	0.041	0.936	0.054
3. R_CSC → Happiness	367.540	245	0.32	0.572	0.040	0.939	0.051

Standardized coefficients are reported.

^*^indicates significance at the 5% level (two-tailed).

Gender, age, marital status, education, household registration and income were incorporated into the statistical analysis as control variables.

**Table 5:** Results of structural equation modeling of cognitive neighborhood social capital on different levels of physical health.

Model	Variable relationship	Levels of physical health	
		(Standardized coefficient)	
		Low level of physical health	High level of physical health
Structural model	Cognitive neighborhood social capital → Mental illness	−0.191^*^	0.081
	Cognitive neighborhood social capital → Happiness	0.679^**^	0.719^**^

Standardized coefficients are reported.

^*^ and ^**^ indicate significance at the 1% and 0.1% levels (two-tailed), respectively.

Gender, age, marital status, education, household registration and income were incorporated into the statistical analysis as control variables.

In terms of mobility purpose, on the basis of the invariance criteria, the floating older adults were divided into two groups, namely “working-oriented” and “family-care-oriented,” and then incorporated into structural equation modeling. Imposing equal constraints on neither the paths from structural neighborhood social capital to mental illness nor those to happiness didn't made a significant difference in the chi-square model compared with the unconstrained model, which indicates that Hypothesis 5.1 and Hypothesis 5.2 were not supported by the results.

As shown in Table 6, when equal constraints were imposed on the path from cognitive neighborhood social capital to mental illness, the chi-square significantly increased compared with that in the unconstrained model. However, the change in the chi-square coefficient was not significant for the path from cognitive neighborhood social capital to happiness. The results presented in Table 7 indicate that the negative effect of cognitive neighborhood social capital on mental illness tendency was observed only for the floating older adults who moved for the purpose of taking care of the family (beta = −0.262, *P* < 0.001), while such an effect was not significant for the floating older adults who migrated for work. Considering the results above, hypothesis 5.3 was proven by multigroup structural equation modeling, but hypothesis 5.4 was not. Mobility motives shape access to social resources. Older migrant workers leverage diverse settings for varied network support, while family-caregiving seniors, constrained by limited mobility and domestic duties, depend heavily on community ties. With constrained choices, a single network intensifies its psychological impact. Thus, cognitive neighborhood social capital more strongly alleviates mental illness propensity in family-caregiving groups.

**Table 6:** Fit index of models of cognitive neighborhood social capital with equality constraints of the main pathway coefficients based on the purpose of mobility.

Paths	*x* ^2^	df	Δx^2^ from the base model	Δx^2^, *P* =	RMSEA	CFI	SRMR
1.Unconstrained base model	388.260	260	–	–	0.042	0.940	0.049
*Constrained paths*							
2. R_CSC → Mental illness	392.430	261	4.17	0.041^*^	0.043	0.939	0.051
3. R_CSC → Happiness	388.570	261	0.31	0.578	0.042	0.941	0.049

Standardized coefficients are reported.

^*^indicates significance at the 5% level (two-tailed).

Gender, age, marital status, education, household registration, income and physical health status were incorporated into the statistical analysis as control variables.

**Table 7:** Results of structural equation modeling of cognitive neighborhood social capital for different purposes of mobility.

Model	Variable relationship	Purpose of mobility	
		(Standardized coefficient)	
		Work–oriented floating older adults	Family–care–oriented floating older adults
Structural model	Cognitive neighborhood social capital → Mental illness	0.019	−0.262^*^
	Cognitive neighborhood social capital → Happiness	0.578^*^	0.630^*^

Standardized coefficients are reported.

^*^indicates significance at the 0.1% level (two-tailed).

Gender, age, marital status, education, household registration, income and physical health status were incorporated into the statistical analysis as control variables.

## Discussion

In China, the multiple roles of aging, urbanization and intergenerational support for cultural traditions have given rise to a new group of floating older adults. They suffer from the dual pressure from aging and mobility at the end of their life course. The new community after mobility is the main place where floating older adults live. Living together based on geographical proximity has led to the development of various connections, interactions and emotional values, which directly impact their mental health (54). This study deviated from the definition of mental health from the perspective of pathology and divided neighborhood social capital into two types to explore its impact on the mental illness tendency and happiness of the floating older adults, which has rarely been investigated in previous studies on aging health. This approach can verify the mental health effects of neighborhood social capital among the floating older adults and further enrich the understanding of its internal mechanisms within the Chinese context.

The results of this study reveal that cognitive neighborhood social capital can reduce mental illness tendency among the floating older adults and significantly improve their happiness. Mobility leads to the disruption or shrinkage of the social network they have established in their places of origin. The mutual support, mutual trust and sense of community belonging gained by establishing new social connections help them rebuild their subjective cognition and identity in an unfamiliar environment, thus improving their mental health. In previous studies, cognitive neighborhood social capital has also been proven to alleviate psychological issues such as post-traumatic stress disorder by providing psychological resilience in disaster scenarios (16–18). This consistency across different situations demonstrates that, regardless of whether individuals are confronted with sudden disasters or enduring mobility pressures, the protective effect of cognitive social capital on mental health remains stable. Essentially, both scenarios entail the breakdown of social networks, the disintegration of daily routines, and the reshaping of identity. These high-stress environments collectively highlight the core role of cognitive social capital (including within neighborhood) in the reconstruction of meaning (38). Further analysis indicates that cognitive neighborhood social capital serves as an intermediary mechanism linking structural neighborhood social capital with the mental health of the floating older adults, which confirms the internal relationship between two types of social capital and mental health in non-disaster scenarios. Cognitive social capital supplies the inner driving force for transformation of structural social capital. Constructing structural social capital is an effective long-term strategy for developing cognitive social capital within neighborhood, which decreases the mental health risk of the floating older adults.

In contrast, the impact of structural neighborhood social capital on mental health of the floating older adults is not the same. On the one hand, it is found that structural neighborhood social capital is positively correlated with their happiness. According to socioemotional selectivity theory, the cognition of time is a powerful driving force for human motivation and emotional experience (55). With increasing age, people perceive the limitation of time, and their behavioral choices are mostly based on maximizing emotions, such as happiness, rather than tools that can result in returns in the future (56). Compared with young people, older adults tend to choose to interact with individuals who can foster rich emotional experiences (57). So neighborhood greetings, chatting with neighbors, and participation in community activities can provide floating older adults with low-cost and high levels of emotional satisfaction and help to improve short-term happiness, especially by alleviating the loneliness and strangeness.

On the other hand, structural neighborhood social capital has no significant impact on reducing the tendency toward mental illness of the floating older adults, which is inconsistent with previous research findings (20, 21). The reason for this discrepancy is the differences in research subjects. Prior research has mainly centered on the local older population, with their structural social capital largely rooted in enduring and stable geographical relationships in the community (21). This high-quality social interaction can not only satisfy emotional needs but also provide deep psychological support. However, the subject of this study is the floating older adults. Their structural neighborhood social capital is more embodied in shallow social interactions. It highlights the differences between them and local residents in terms of language, culture and habits and aggravates their psychological experience as “outsiders”. If this difference cannot be adapted, adjusted or reduced by the floating older adults, it is likely to turn into deep and lasting emotional problems, including psychological alienation, loneliness and meaninglessness. These deep negative emotions can be directly intervened in and transformed through cognitive social capital. In comparison, structural social capital, such as social frequency and network scale, is difficult to achieve.

Heterogeneity analysis is also one of the contributions of this study and has rarely been considered in existing research. Instead of selecting normalized variables such as age, income, and education (32, 33), this study focused on the unique dual attributes of aging and mobility characteristic of the floating older adults, and physical health and mobility purpose were chosen for heterogeneity analysis. It is conducive to revealing that the operation of neighborhood social capital and its impact on mental health are based on specific situations (42). The results show that for floating older adults with poor physical health, structural neighborhood social capital has a stronger positive effect on their happiness, whereas cognitive neighborhood social capital has a greater role in alleviating mental illness tendency. Cognitive neighborhood social capital has a stronger negative effect on the mental illness tendency of family-care-oriented floating older adults than on that of older migrant workers.

The dual attributes of aging and mobility make the floating older adults unique in terms of social ability and resource dependence. From the perspective of aging, the decline in physical health limits their daily activities and spatial accessibility (58), forcing social interactions to be confined to the community, resulting in functional dependence on neighborhood networks. From the perspective of mobility, the floating older adults who move for family caregiving purposes are heavily engaged in household work and the commitment to family responsibilities. Their social roles are confined to the private sphere and the reconstruction of social networks is highly dependent on the community, which is a space geographically close to the family. Older migrant workers generally have rich mobility experience, strong adaptability and diverse social situations (mainly workplace and community), which make it easier and more possible to make new friends in unfamiliar environments and to obtain access to a certain degree of heterogeneous network resources (44). Functional decline and role constraints jointly weaken individuals' access to alternative resources, thereby increasing the dependence on neighborhood social capital. Therefore, the positive effect on mental health is reflected mainly in those with relatively poorer physical health and motivated by family caregiving needs.

In summary, this study has made several significant theoretical contributions. First, it broadens the application of positive psychology to the mental health research of the floating older adults, beyond the conventional “problem-focused” paradigm. Second, within the framework of China's mobile population health, this study advances social capital theory by clarifying the connection between structural and cognitive forms of neighborhood social capital and mental health in non-disaster contexts. Third, considering the dual characteristics of the floating older adults, the study identifies physical health and mobility purpose in shaping neighborhood social capital's impact, addressing “for whom social capital works” and supporting resource substitution theory in the field of mental health research.

Based on the above empirical research results, this study has the following policy implications. First, to accommodate the intergenerational care needs of the floating older adults, community public facilities such as fitness centers and parks should be renovated into intergenerational shared spaces. By organizing activities centered on sports and entertainment as entry points, community engagement among the floating older adults can be boosted, thereby fostering the development of informal social networks. Second, build a “story experience sharing” platform and institute a regular mutual aid system, exemplified by one-on-one partnerships between local and non-local older adults, to foster deeper trust and belonging among the floating older adults within the community. Third, provide targeted support for high-dependency groups. Deliver home-based community services (e.g., health check-ups, companionship visits) for those with poor physical health. Establish peer support platforms (e.g., childcare experience-sharing groups) for family-care-oriented floating older adults.

### Limitations and future research

This study has several limitations which future research can optimize. First, since there are no panel data specifically for the health of the floating older adults in China, this study could use only cross-sectional data for analysis. Second, because this study only collected data from three cities in Guangdong Province, it is necessary to verify if its conclusions can be generalized to other regions of China. Apart from that, this study primarily focuses on the impact of neighborhood social capital on mental health. Although some key demographic and sociological variables were considered, some potential influential variables such as mobile duration and community type were not included in the current analysis to keep the model concise and avoid over-interpretation. Finally, the KMO and Cronbach's alpha values of some scales, such as social wellbeing and cognitive social capital, were lower than 0.8, which meant acceptable but not optimal. This implied that measurement was insufficient in fully capturing the distinctive experiences of populations undergoing social transition. Moreover, evaluating abstract concepts imposed cognitive challenges on the floating older adults, who possess varying levels of education and cognitive abilities. In light of these factors, future research should focus on developing customized tools tailored to this specific demographic.

## Conclusions

This study aims to explore the relationship between neighborhood social capital and the mental health of the floating older adults in China. Based on the empirical analysis, the hypothesis testing results are summarized as follows: structural neighborhood social capital is positively correlated with happiness but has a limited effect on mitigating mental illness tendencies (H1 partially supported); cognitive neighborhood social capital significantly reduces mental illness tendencies and increases happiness (H2 is supported); it plays a mediating role in the relationship between structural neighborhood social capital and mental health (H3 is supported). Physical health and mobility purpose are key moderating variables that affect the relationship between neighborhood social capital and mental health (H4 and H5 are partially supported).

There are two key empirical findings. First, the two kinds of neighborhood social capital have different effects on the mental health of the floating older adults. Cognitive neighborhood social capital significantly reduces the tendency toward mental illness and enhances happiness through its impact on meaning restructuring. In contrast, structural neighborhood social capital is mostly limited to superficial interactions, which easily leads to an outsiders' experience for the floating older adults. It has a relatively weak direct impact on their mental health, but can indirectly exert a positive effect by promoting cognitive neighborhood social capital. Second, neighborhood social capital has a stronger protective effect on the floating older adults with lower physical health and those who move due to family caregiving. The benefits of neighborhood social capital on mental health are amplified for vulnerable populations with higher welfare dependency.

This study makes three theoretical contributions. It surpasses the traditional disease-centered perspective, incorporates a positive psychology perspective, and emphasizes a dual focus on mental illness tendencies and happiness. Moreover, it deepens the understanding of how different types of neighborhood social capital influence mental health, verifying that structural social capital serves as the cornerstone for fostering cognitive trust and reciprocity, ultimately improving health outcomes. Finally, the heterogeneity analysis in this study provides important theoretical insights for community-based interventions, highlighting the necessity of implementing precise support strategies for welfare-reliant subgroups among the floating older adults.

## Data Availability

The anonymized raw data supporting the conclusions of this article contain personal health information of the floating older adults. Due to ethical and privacy restrictions, the data are not publicly archived. Anonymized data may be obtained from the corresponding author upon reasonable request, subject to approval by the institutional ethics committee.
